# Indents

**Published:** 1907-12-15

**Authors:** 


### INDENTS.

☞Brief Articles of Techinical or Scientific Value will receive notice and comment. Contributions invited.

**Low-Fusing Alloy.** In answer to a request for a low-fusing alloy, one which will melt below the boiling point of water, the following may be used: Tin, 2 parts; lead, 4 parts; bismuth, 7 parts; cadmium, 1 part. This fuses at about 160 degrees Fahrenheit.

**To Make a Hard Model.** When for any reason it is desired to make an especially hard model, a small quantity of Portland cement, mixed with the plaster, will accomplish the purpose. About one part of the cement to twenty of the plaster is a good proportion.—*Western Journal.*

**Decay of Children's Teeth.** I believe that the great cause of the decay of children's teeth, aside from neglect, is the lack of use. If children were taught to properly masticate it would go a long way towards keeping the mouth in good order and the exposed surfaces polished.—Burton Lemly, *Pacific Dental Gazette.*

**Soldering Aluminum.** Flux: 80 parts stearic acid, 10 parts zinc chlorid, 10 parts tin chlorid. Any ordinary soft solder can be employed, but the best is that composed of 80 parts tin and 20 parts zinc. Cleanse the article to be soldered and pass through the flux; solder in the usual way.—*Quyrterly Circulyr.*

**The Clasp.** With a band more or less broad, we are always likely to have food and moisture retained between the band and tooth. It is a good idea to use thin 16k. gold wire bent on itself in the form of a loop and carefully fitted just above and below the broadest part of the tooth. By this method it is possible to get as powerful a clasp as is necessary with considerable springiness, and yet only a wire line of actual contact, which renders it almost self-cleansing.—Mr. Hey, *British Journal.*

**To Obtund Sensitive Dentin.** Flood the cavity with pure carbolic acid, and wait a minute or so; then put into the cavity a little finely powdered peroxid of sodium without wiping out the acid. This causes some pain. Add a few more grains and commence to carefully excavate. If the rubber dam is not used, care must be taken of the gum.—HENRY I. MOORE, Frankfort, Germany, *Dental Review.*

**Baby has Teeth at Birth; may Become Rival of Roosevelt.** An infant son born to Mr. and Mrs. I. E. Coddier, Mishawaka, Ind., September 10, is likely to become as famous for his teeth as President Roosevelt. The attending doctor and nurse, when looking at the infant, were amazed to find that it was already provided with eight teeth, four in the upper and four in the lower jaw. They were protruding for a safe distance, and resembling pearls set into the tender gums of the infant. The case is one of the rarest ever recorded.—*Digest.*

**Putrescence in Pulp Chamber or Root Canal.** Isolate the tooth, wash its interior carefully with copper-sterilized water, partially dry it and place in the center of the pulp chamber a crystal of mono-chloro-acetic acid and seal hermetically with cement. After a few days open with antiseptic precautions—that is, do not allow saliva, hydrant water, or an unsterilized broach or instrument to enter the root canal, or go through it to push any foreigu matter into innocent tissues. The worst case of putrescence will yield after two treatments at intervals of one week.—A. W. Harlan, *The Dentist's Magazine.*

**Taking Impressions.** I have one little suggestion to offer in the taking of impressions—particularly in those cases that are extremely sensitive and easily nauteated—that I have found useful and helpful, and that is so sponge the mouth with hydrogen dioxid. After thus having cleaned all the mucous surfaces apply a solution of eaucain to the whole palate. That will enable one to take an impression in the most exaggerated cases of palatal sensitivity.—T. B. Hartzell, *Texas Dental Journal.*

**Local Anesthetic for Pyorrhea.**

| | |
|---|---|
| Chloroform | ounces 1 |
| Cocaine | grains 20 |
| Oil Cloves | drops 8 |
| Oil Cassia | drops 8 |
| Menthol | grains 8 |

Before removing deposits from roots saturate pellet of cotton with solution, crowd gently into pockets and allow to remain for a few moments. Take care to keep cork in bottle, as the chloroform will evaporate rapidly.—J. D. PATTERSON, in *Western Journal.*

**Silver Nitrate in Pyorrhea.** After the surgical operation we find the necks of the teeth extremely sensitive. Cold air hurts, and it is extremely disagreeable for the patient; and there is nothing which gives relief like nitrate of silver. I keep a fresh saturated solution and apply it by means of shreds of cotton wound on an orange wood stick, protecting the gums and mouth with cotton rolls. The stain will wear off in a few weeks, and the teeth will have recovered from sensitiveness.—Dr. J. D. Patterson, *Era.*

**Pyorrhea Pockets.** In all cases of deep pockets, difficult of access, simplify the work by previously packing with gauze saturated in 25 per cent phenol—sulphuric acid, leaving it for twenty-four hours when the gum will be crowded away from the tooth neck enabling you to see to a great extent exactly what you are doing and to scale the root without much pain or laceration of tissue.

This drug has a tendency to soften the calculus, making its removal much easier.—Elgin MaWhinney, *American Dental Journal.*

**Vulcanizing Rubber.** I believe that the majority of rubber dentures are vulcanized at too high a temperature and in too short a time. A denture should be vulcanized at 280 or 290 degrees—depending upon the rubber—for three hours after the vulcanizer has reached that point. In that time a thick lower denture can be vulcanized solid; you can saw right through the mass and polish the cut surface, but you cannot do that if vulcanized at 320 degrees for fifty-five minutes approximately.—Dr. Gritman, *Dental Cosmos.*

**Sensitive Dentin.** Some time ago Dr. Fossume spoke of the use of "Formalin" for controlling the sensitive spots at the necks of the teeth that are sometimes so troublesome. At that time I questioned its safety, but since trying the method I have found it so satisfactory that I wish to speak of it at this time. The "Formalin" should be used full strength and it must be kept away from the gum. A very short application is sufficient to control entirely a very large proportion of these cases.—H. W. Gillett, October, 1906, *Journal.*

**Treatment of Sensitive Palates prior to the Taking of Plaster Impressions.** In prosthetic work, when your patient has a tender palate and is bothered with retching, to stay such trouble while taking an impression, paint on palate a 20 percent. solution of potassium bromid with a camelshair brush, or gargle 5 to 15 gr., or both. In extreme cases give ten gr. at night, repeat after breakfast and again, half an hour before impression is taken, give ten to fifteen gr. It greatly diminishes the sensitivity of a tender palate.—H. E. Davis, *Dental Era.*

**To Re-Bake an Inlay.** If for any reason you desire to re-bake a porcelain inlay after you have removed the platinum matrix, make an investment of powdered soap stone and thin shellac varnish, while investment is fresh imbed the inlay cavity side down until flush with margins. Dry slowly, and you may fuse as high as you like with no change to margins or shape. This would probably not do for low fusing porcelain, because the shellac would not all burn out and a discoloration would develop. The soap stone powder may be made by scraping a fresh mechanic's crayon.—J. M. Evey, in *Tri-State Record.*

**Echinacea.** I am greatly prejudiced against so much hypodermic medication. I think it is greatly overdone, and I believe there is much danger of infection. I was very reluctant to let a dentist inject cocaine into my gums, as he desired to do for the extraction of nineteen or twenty teeth. His probing however, was very severe and left me with very sore gums. I immediately applied to the gums full strength echinacea, and kept it in contact for some time. The result was highly satisfactory; the soreness was completely relieved, and all unpleasant results abated.—Geo. Hare, M. D., in *Ellingwood's Therapeutist.*

**Inspection of School Children's Teeth.** Malden, Mass., looks after the teeth of its school children. By a State law, cities and towns are required to provide for a medical examination of the pupils, but Malden goes a step further. Its school board is sending dentists around to the various schools for the purpose of making an examination of the teeth. If they are found in bad condition and require attention, a slip showing the remedies needed is sent to the parents, offering the suggestion that the matter be attended to at once. Already some of the schools have been visited with beneficial results, and many of the parents are loud in their praise of the action.—*Gloucester Times.*

**Cutting Out Fissures.** In cutting out fissures in the molars and bicuspids, instead of using fissure burs, use half-inch knife-edge carborundum stones with screw mandrel on right angle; turning the right angle either to the cheek or tongue, whichever will be necessary to prevent the stone from jumping. With the back of the mouth-mirror toward the stone, hold the cheek and tongue out of the way. After cutting the fissure sufficient in depth and width, bevel the edge by leaning the side of the stone against it; and at the same time undercut the opposite side. Use stone in the hand piece in cutting out pallatal fissures, and cross-cutting the fissure in top of the lower molars. The green colored stone is not so easily broken, and will outwear the others. After they are worn down small, use them in cutting out short fissures. I have not used a fissure bur in ten years. The stones cut much faster, smoother, with less pain and less expense.—B. T Radcliff, in *Brief*.

**Setting Inlays.** Many of the failures of cemented fillings or inlays are due to indifference in the operation of cementation. One of the advantages of large inlay restorations, as compared with crowns, is that the rubber dam may be applied, the tooth thoroughly dried and kept dry until the cement has thoroughly crystallized. A slow-setting cement is always preferable. The method of cementation which I use for all inlays, porcelain and gold, is as I will now describe:

Apply the rubber dam, thoroughly cleanse the cavity; make such undercuts as may be had without endangering the strength of the tooth; apply slow-setting Harvard cement, rubbing it thoroughly into the cavity with a ball burnisher, and on the cavity side of the filling; carry the filling to place, manipulating it with pointed instruments to be sure that it is perfectly seated, and allow it to remain under the rubber dam for forty-five minutes.—S. H. McAfee, in *Cosmos*.

**Tropa-Cocain.** The effect of tropa-cocain on the nervous system, as well as upon the respiration, is analogous to cocain except that it is much less dangerous. It produces anaesthesia more rapidly, more pronounced and of longer duration. As an ideal antiseptic local anaesthetic I use the following formula exclusively:

| | |
|---|---|
| Tropa-cocain | gr. xvj |
| Chloreton | gr. x |
| Spt. glonoin | gtt. xvj |
| Water | ℥ij |

Boil the water and then add the chloreton, ten grains, or as much as it will dissolve, for it is soluble only to the extent of 1%
—B. L. Kissel, Dental Era.

**How Often Should Orthodontia Patients be Seen.** Twice a week is often enough for almost any case at any time, and a large share of the time once a week is all that is really needed. It is always advisable to see patients twice a week where possible to avoid loss of time from appliances getting out of order, for no matter how carefully fixed, things will go wrong at times, and the patient may not always be aware of it either, resulting in much loss of time. Many cases have been successfully treated with one visit a week, and still others with one visit every two weeks. This latter does not give the operator a chance to do himself justice, however, and is not to be recommended. Twice a week is often enough to be safe, and not too often to be burdensome to the patient.
—W. J. Brady, in *Western Journal.*

**Dentist Restrained.** Dr. Fred. E. Roby, a dentist, is restrained from doing business at his office in Boylston street, under a decision of the Supreme Court. By selling out his old business and its good will, that court holds that a professional man, like a dentist, impliedly agreed not to re-enter practice again near enough to the old stand to impair the business.

Dr. Roby was formerly in partnership with Dr. Lewis T.

Foss, having an office on Court street. Roby sold out his interest to Foss. Three years later Roby opened an office on Boylston street. Foss, who had formed a partnership with another doctor, sued for an injunction to restrain Roby from practicing. Judge Hardy, of the Superior Court, dismissed the bill and the complainants appealed.

The full bench held that the complainants were entitled to an injunction restraining Dr. Roby from practicing his profession in Boston. They may get damages, too, for breach of contract. —*Boston Globe.*

**Pressure Anesthesia.** Some time ago, seeing Dr. Tuller's suggestion in regard to pressure anesthesia in the case of an aching tooth, I have upon several occasions made use of the method with most gratifying results. I find it most useful in those cases in which the patient is suffering from an exposed and congested pulp, in which cases I apply the anaesthetic quite freely to the bottom of the cavity, and seal in with permanent gutta percha. The pain immediately following the application is sometimes quite severe, but in a few minutes this ceases, together with the pain which originally existed. The same treatment has proved effectual in several cases when the pulp was not entirely uncovered. Under such circumstances the application of the cocain must not be made until the cavity and decayed dentine are thoroughly dried. I wish to add that my experience has been most satisfactory, and I favor this as a means of relieving common toothache when many other methods will fail.—X. in *Brief.*

**How to Obtain a Correct Bite.** Perhaps the idea is not a new one at all, but it was to me. Take, for instance, a full upper case—of course it will be the same with any other case—take an impression, run the cast, wax up the case, set on the six anterior teeth by guess, tack on a piece of wax over the ridge where the posterior teeth should be, about the proper height. Then warm the piece of wax over the ridge, try the piece in the mouth, and let the patient bite down, and that will make prints of the lower teeth. You can look in the mouth and see whether the patient closes naturally. Next take an impression—preferably with modeling compound—of the lower teeth, and after preparing the cast, set the latter in the print in the wax that was made by the lower teeth, and fasten both to the articulator. The advantage over the ordinary method of obtaining the bite is that one can readily see if the patient bites correctly. It takes no longer to take the bite in this way.—T. A. LIFSEY, *Dental Summary.*

**Beveled Walls.** If a carpenter or joiner were to take a piece of board that he was joining, I will say this floor, and saws it square across and butts the ends together, he is making a butt-ended joint; and the closeness with which he is able to butt that joint together is exactly in proportion to the amount of personal force he can employ. If he would take that board and bevel one end in one direction and the other in the opposite direction, he could then close the joint with at least fifty per cent less crevice than were he to butt the two together.

We want to apply that principle to cavity formation so that in cementing the inlay in to place we are able to press it into the cavity with the minimum of crevice,—that is, we are able to crowd out the cement to the greatest extent possible to do. A flat floor and right angles mean a butt-ended joint. In contradiction I would say that all the cavity formation should be as nearly as it is possible to make it so that when the inlay enters the cavity it enters on the wedge principle, diverging just enough from parallel walls so that we eliminate the thickness of the platinum matrix. —*Dr. Reeves.*

**Dental Clinic in School.** United States Consul E. T. Liefeld reports that on April 22 a municipal school dental clinic was opened in the German City of Frieburg, the operations of which he thus describes: "The dentist at the head of this school clinic examines all the children in the city, both in their homes and in the public schools. A report on such examinations is sent to the parents, who are asked to send their children to the school dental clinic for dental free treatment. Those children having ten or more poor teeth are first treated, an exception being made in a few higher classes where those with only slight defects are to be treated, so that they will leave the public schools with sound teeth. After these worst cases have been attended to, all other children with defective teeth are to be treated, the younger ones given preference. The treatment of the teeth includes extraction, filling, crowning, etc. There is no actual instruction in dental hygiene, but at the opening of the dental clinic the teachers explain its objects and workings to the children. The tooth report card contains on the reverse side instructions as to the care of the teeth.—*Dental Brief.*

**Anesthetizing Inflamed Pulps.** As a rule, the most extreme tenderness of the dental pulp is at or nearest the point of the lesion. Having attempted once or twice first to fully open, and secondly to anesthetize the pulp at the point of the lesion, but having failed, I then approached the pulp from another flank, *i. e.*, drilling through the enamel and dentine with a double-bevel sharpened No. 1 rose bur. This procedure is less painful than would at first be supposed. Having reached the pulp at such a point, the medicinal preparation is readily absorbed, and the pressure that may be exerted is less painful. One slight application of pressure is often sufficient to enable one to return to the original cavity, which only a moment before was almost too painful to approach, and to open it painlessly. The medicinal preparation I use is pure cocaine hydrochlorid, crushed and made into a paste by the addition of oil of cloves This is applied on the pulp, and is followed with cotton saturated with extract of Indian hemp, and the whole is placed under a compress of black unvulcanized rubber. The congestion ot the pulp being relieved, I carry up the root, it found necessary, a very mild solution ot cocaine, say of one to two per cent strength.—G. A. Englert, in *Cosmos*.

**Don't Promise.** A prolific cause of trouble with artificial dentures is the rash promises made by the dentist when the work is started; or if not promises, at least neglect to warn the patient of the difficulties to be encountered before the plate will be worn with comfort and satisfaction. It is so common to hear a patient say, "Well, I am glad to be rid of those old snags at last; I will have a set of teeth now that won't ache, anyhow." It is a wise precaution to assure the patient, at this time, that while the new teeth will probably not ache, they will give trouble of another kind that may make the wearer wish, for a time, for the old "snags" back again. A plain explanation of the difficulties to be encountered, and the time that must elapse before the plate will cease to annoy, may save a lot of explanation later, at a time when it will not be accepted so readily. If the plate, as sometimes happens, gives little or no trouble, so much the better. You have done better than you promised and left a pleasant impression. It is always better to have work of any kind prove more satisfactory than promised, than to have it prove less. An artificial denture is but a poor substitute for the natural organs at best, and it is unsafe to promise very much in the way of service. —*Western Journal.*

**Mixing Cements.** The granular effect sometimes noticed in cements is caused by trying to mix too large a portion of powder with the liquid at one time. The proper method of mixing is to place your liquid and powder at opposite ends of the slab, and mix a small portion of the powder with all the liquid thoroughly, then add powder a little at a time until it has reached the proper consistency. The discoloration noticed after mixing is almost always due to use of an improper spatula. A spatula should be of bone or be nickel plated. The nickel prevents the steel or iron from coming in contact with the phosphoric acid; for when steel or iron comes in contact with this acid it forms a ferrous phosphate which will discolor and affect the quality of the cement. To get the best results a dropper should be used in taking liquid from bottle. The bottle should be thoroughly shaken before used. The powder bottle should be kept corked so that the powder will not absorb moisture; a bone or nickel spatula should be used, a small amount of the powder should be thoroughly incorporated with all the liquid and the balance of the powder added in small quantities and each portion thoroughly mixed until the proper consistency is secured.—T. E. PURCELL, in *Western Journal.*

**Evils Incident to Use of the Dam.** In the following operations the dam may be omited for hygeinic reasons, comfort of patient, economy of time, clearer view of the case, and for other advantages not here mentioned: (1) Opening tooth for ventilation of abscess, evacuation of pus, cleansing of chamber, canals, etc. (2) Removal of putrid matter or whatever should be removed from cavities or canals already exposed to the fluids of the mouth. (3) Cleansing of enamel and removal of filth and decalcified dentin—in short, the greater part of the preparation of all cavities. In these cases a stream of tepid water cleanses the field of operation, dissolves or absorbs fetid and volatile matter, removing the whole to where it can no further offend and incidentally cleansing the mouth to the advantage of patient and operator. A self-filling syringe renders this use of water quite convenient. Where a dam is used the foul, infectious, poisonous contents of the cavity are evaporated by blasts of air, and when cut to suit are puffed out to the immediate contamination of the air breathed by patient and operator. Filthy dust of this kind once raised is distributed to everything in the room. It is not easily removed and so remains a menace to the health of operator and patients. It would, therefore, seem well to avoid the dam and dust-blowing where it is not essential to correct work. W. C. Gowan, *Digest.*

**Instantaneous Relief in Cases of Blind Abscess.** Instantaneous, and often permanent relief from pain following infection from a pulpless tooth may be obtained by the use of equal parts absolute alcohol and water. Whether the pain is caused by an upper or lower tooth, the treatment is equally effective.

The preparation can best be used with what is known as a watch-case atomizer. Fill the atomizer with the solution, then, having the patient recline the head a little, place the nozzle of the atomizer into the nostril on the side where the trouble is; spray the solution back into the nostril twice and the pain will be relieved immediately. In some cases there will be a slight amount of gaging, and the solution will come out through the mouth, but this discomfort is only temporary.

If the pain should return within fifteen minutes, a stronger solution, say seventy-five per cent. alcohol and twenty-five per cent. water, may be used, and the treatment repeated; but usually the fifty per cent. solution will be sufficient, and will permit you to open up the tooth and give the patient relief.

The watch-case atomizer may be obtained from almost any large drug house. If you can not get one, use any atomizer that will force a spray far back into the nose.—DR. I. E. KEEFE, in *Items of Interest.*

**The Retention of Upper Dentures.** An article in a recent JOURNAL upon the retention of upper dentures, while correctly ignoring the use of vacuum cavities, overlooks an important feature, and it, is very generally overlooked or ignored.

Every dentist is aware that the center of the palate, in ninety-eight per cent. of jaws, is hard and unchangeable, but forgetting it is the only portion of the jaw that does not change, while the alveola ridge does change so that it is only a question of time when the plate, instead of resting as it should, upon the ridge, is resting and rocking upon the hard center. The remedy for which is to cover the hard center, in case of a metal plate, with a "relief," using the thin base plate wax, leaving the margin thin or flush with the model. This should extend nearly to the top of the ridge and as far back as possible, but the plate should extend about one-fourth inch beyond. Plates can be worn farther back than usually made, almost on a line with base of the tuberosities. This is the only change I make in a model.

In vulcanite work, scrape the impression or finish out of the plate. It cannot be too strongly impressed upon the mind of the patient that vulcanite is unsuitable for permanent work on account of its retention of undue heat, causing absorption of the ridge. This is a matter I have been observing ever since the introduction of vulcanite, nearly fifty years ago.—L. P. HASKELL.

**Extracting for Regulating.** Some years ago a prominent Chicago dentist called at my office and said: "Doctor, when you have cuspids that are erupting through the gums above, and with little or no spaces between the laterals and first bicuspids, you have to extract teeth, don't you, to get them into the arch?"

I explained to him and showed with many plaster casts that, however irregular the teeth, however bunched, maligned or malposed, they could always be placed in their respective places in the arches and in normal occlusion; therefore, so far as the relations of the teeth to each other are concerned no dental malposition should be taken as a basis for extraction. The only excuse then for the extraction of saveable teeth, must be that it is inexpedient or impossible to correct their positions in that way without producing facial protrusion. In nearly all locally caused malpositions of the teeth in immature arches, the final development of the jaws and general growth enlargement of the features, demand all of the teeth and their sustaining alveolar arches to harmonize facial relations. To remove one or more of them under these conditions will inevitably produce its effect. The effect upon normal arches that would be otherwise harmonious in size and relations, is invariably an abnormal contraction of the arch so effected, and the ultimate forcing of the teeth of the opposing arch into malalignment and malocclusion which frequently results in a facial deformity.—C. S. Case, in *Review*.

**Non-Corosive Sterilizer for Dental Instruments.** Requirements: In form, liquid; in action, rapid, certain, penetrating, bland, volatile, cleanly, uncontaminating, non-toxic, non-irritant, antiseptic, germicidal, portable, inexpensive, analgesic, easily prepared, convenient to apply to each instrument each time used and non-corroding.

R Specific echafolta (Lloyds) ........ ℥jss

Alcohol deodorized ........ ℥ss

Glycerin Pur. ........ gtts.x

Ol. Rose Geranium ........ gtt. ij

Mur. cocaine (coarse crystals) ........ grs. xxxvij

Acid carbolic (crystals) ........ grs. xx

M. Sig. as directed.

Procure a glass bonbon dish three inches in diameter by one and one-half inches in height; sides oval; in use pour off compound from well corked bottle enough to cover a bur-head, drill, excavator or searcher point, and before or after using, either dip in the compound each time either is used and at night before laying away. Forceps points wet with the lotion will become instantly sterilized. Two or three drams of the compound will last a month if evaporation is met by the addition of alcohol and the dish is kept covered when not in use. A piece of thick rubber-dam makes a good cover. The dish and two ounces of the compound need not cost more than $1. I have used the preparation for years and know its efficiency. Its value is beyond price.—A. C. Hewett, *Dental Review*.

**An Extraordinary "Mechanical Face."**

Dr. Delair has presented to the members of the French Academy of Medicine a man who, in consequence of an accident with a fowling-piece while out shooting, had his chin, a portion of the lower jaw-bone, his lips, a portion of his tongue, the whole of his upper jaw, and his nose blown away. The missing part of his face has been replaced mechanically, and the medical men present expressed astonishment at the marvellous ingenuity with which the work has been accomplished.

The mechanical face consists of four pieces. The first piece consists of a silver groove, into which are fixed the lower teeth. This is fitted to a dental apparatus of tin, into which are fixed the remaining teeth. The second piece consists of a dental apparatus in vulcanite and gold for the upper row of teeth. This is fixed to two small horns, which fill the nasal cavity. It also fills up the right sinus, which was smashed in. This apparatus is fitted with nine teeth. At the rear is a piece of gold mechanism, which is used to fasten or hook on the face-piece.

The third piece of the mechanical face consists of the chin and lower lip. These are in india-rubber, and painted to resemble Nature. Over the chin a false beard is fixed. At the back of this portion of the mechanism are a couple of small bolts, which pass through the holes of the dental piece and fix the chin and lip to the artificial lower jaw and gum.

The fourth and last piece of the apparatus consists of the upper lip and the nose, also in india rubber, painted, and to which is attached a false mustache. At the rear are two small clasps, which automatically fix on to the upper dental piece and jaw.

The man is able to masticate his food with comparative ease, and from a distance of a few feet the appearance of his face is quite lifelike.

.... The man is able to take his artificial face to pieces himself, and he washes it in soap and water every day.—*Dental Surgeon.*

**Neck Broken by Dentist.** George Davis, 38 years old, 32 West Madison Street, died to-day at the county hospital from a broken necd, suffered while he was having a tooth extracttd by a dentist.

The case, it is declared, is without precedent in the history of the hospital. Davis died after suffering an attack of paralysis in his right arm, and when an examination was made it was found that his neck had been broken. Efforts were begun at once by Deputy Coroner Davis to learn the identity of the dentist who treated the man.

Davis was taken to the hospital Monday night. At the time he complained of an injury to his neck, and he was closely questioned.

Dr. A. B. Eustace conducted the examination. The physician said: "Davis told me that he had had a molar tooth extracted by a dentist two weeks ago. He said it was a big tooth and that the dentist had trouble taking it out. The dentist was forced to jerk at the tooth several times before it was finally withdrawn, but at the time Davis declared he felt no paid in his neck. Later, he said, he suffered an attack of paralysis in his right arm and was troubled with a pain in his spinal column.

"The pain later went to the cords of his neck. I made an examination after he told me his story and found that his neck was broken and that the attack of paralysis and the pain in his spine was due to the injury. I was then convinced that the man's neck was broken while the dentist was jerking at the tooth, when he declared that he had not met with any accident and was unable to account for the injury except in the visit at the dentist's office.

Davis' body was taken to the county morgue, and, to determine the exact cause of death, Coroner's Physician Dr. Warren Hunter, made a post-mortem examination. The broken neck was found.

Davis rallied for a time at the hospital, but later became unconscious and died without regaining his senses. The

Desplaines Street police were called upon to discover the identity of the dentist who extracted the tooth.—*American Journal.*

**Codrenin.** Codrenin is the name adopted for a solution of Adrenalin and Cocaine, just placed upon the market by Parke, Davis & Co. Each fluid ounce contains:

Cocaine hydrochloride, 4 3-5 grs. (1 per cent.)
Chloretone, 2 1-4 grs.
Adrenalin Chloride, 1-12 gr. (1,5000).

The two active ingredients are dissolved in a physiological salt solution that contains sufficient Chloretone for preservation.

The local anesthesia produced by cocain alone is very fleeting, for the reason that the drug is rapidly absorbed into the general circulation, frequently with toxic effects, owing to the comparatively large quantity that must be injected. To overcome these difficulties the combination of Adrenalin with the anesthetic has been extensively resorted to in recent years. When Adrenalin is injected into the gum or tissues it has the immediate effect of rendering the part almost entirely bloodless, a condition which lasts for a considerable time. In other words, the circulation of the blood in the part is practically suspended. As might be expected, the injection of Adrenalin in conjunction with an anesthetic localizes the action of the latter by preventing its immediate absorption into the blood; this local anesthesia is not only secured by the use of a much smaller quantity of Cocaine, but it lasts for a longer time, while the chance of producing untoward symptoms is very much reduced as the small amount of anesthetic injected and its very slow absorption. It may therefore be said that the addition of Adrenalin to Cocaine greatly increases and prolongs the anesthetic effect of the latter, so that weak solutions act as well as, or even better than, stronger solutions without the Adrenalin.

Laryngologists, rhinologists, and all who have occasion to perform minor surgical operations will find Codrenin of great service, accomplishing for them what it does for the dentist, namely, the prevention of pain and the mitigation of bleeding.

For minor surgical operations the solution may be used undiluted, but when a large area requires to be anesthetized it may be diluted with four volumes of physiological sodium chloride solution. Dilutions should be made as desired for use, and not in the original bottle. The bottle should be promptly re-stoppered after use, and kept in a drawer or cupboard protected from light.

Codrenin is marketed in one-ounce glass-stoppered bottles only.—*Therapeutic Notes.*

**Artificial Dentures Should Duplicate Nature.**

A full upper and lower denture should, if correctly constructed, duplicate the conditions found in Nature. That is, there should be numerous and widely distributed points of contact between the two sets of teeth, so that when the movements incident to mastication are performed, the two sets will be held in place, and there will be no tipping of the plates upon the alveolar ridges. In setting up the teeth this condition is more easily arrived at, if the process of eruption of the natural teeth is kept in mind and imitated. The appearance of the plate in the mouth and its harmony with the features, depends almost entirely upon the arrangement of the upper incisors, cuspids and bicuspids, and it is always advisable to try the upper plate in the mouth of the patient after these teeth have been arranged and before proceeding further. When assurance is had that these teeth are correctly set, the lower second bicuspids are next mounted so that their cusps come into the interspace between the upper bicuspids. The lower first molars follow, their height being gauged by the overhanging distal surface of the upper second bicuspids; and their positions are tested by lateral movements of the articulator, and they are set so that they will maintain contact, but not interfere with that of the bicuspids. Then the upper first molars are added, and their positions tested in the same manner. After these come the lower second molars and the corresponding upper teeth. When they are all mounted, they should all remain in contact during lateral movements of the articulator.

The lower first bicuspid, coming as it does between the upper first bicuspid and cuspid, is free upon the mesial side of its grinding surface. The cuspids and incisors, being added in pairs, should be kept out of the way of the articulation of the molars. If they should be too wide to fill the space, they should be exchanged for narrower ones. The teeth already set should on no account be moved backwards to accommodate them. Frequently the cusps of the teeth require grinding before they will articulate as they should and when the need for it is apparent, there should be no hesitation about applying the carborundum wheel. When a correct articulation is thus secured, and the denture is finished, it will be found to be one from which the patient can derive all possible benefit so far as the process of mastication is concerned.

This matter has passed the theoretical stage. A number of dentists have adopted the principles here laid down in practice with the best results. And it may be said that their understanding and adoption will enable those who use them to do better service to their patients than has heretofore been possible.—Dr. Snow, in *Dentist's Magazine.*

